# Chemokine CXCL13 acts via CXCR5-ERK signaling in hippocampus to induce perioperative neurocognitive disorders in surgically treated mice

**DOI:** 10.1186/s12974-020-02013-x

**Published:** 2020-11-08

**Authors:** Yanan Shen, Yuan Zhang, Lihai Chen, Jiayue Du, Hongguang Bao, Yan Xing, Mengmeng Cai, Yanna Si

**Affiliations:** 1 Department of Anesthesiology, Nanjing First Hospital, Nanjing Medical University, Nanjing, 210006 People’s Republic of China; 2 Jiangsu Key Laboratory for Design and Manufacture of Micro-Nano Biomedical Instruments, School of Mechanical Engineering, Southeast University, Nanjing, 211118 People’s Republic of China

**Keywords:** Perioperative neurocognitive disorders, Hippocampus, CXCL13, CXCR5, p-ERK

## Abstract

**Background:**

Perioperative neurocognitive disorders (PNDs) occur frequently after surgery and worsen patient outcome. How C-X-C motif chemokine (CXCL) 13 and its sole receptor CXCR5 contribute to PNDs remains poorly understood.

**Methods:**

A PND model was created in adult male C57BL/6J and *CXCR5*^−/−^ mice by exploratory laparotomy. Mice were pretreated via intracerebroventricular injection with recombinant CXCL13, short hairpin RNA against CXCL13 or a scrambled control RNA, or ERK inhibitor PD98059. Then surgery was performed to induce PNDs, and animals were assessed in the Barnes maze trial followed by a fear-conditioning test. Expression of CXCL13, CXCR5, and ERK in hippocampus was examined using Western blot, quantitative PCR, and immunohistochemistry. Levels of interleukin-1 beta (IL-1β) and tumor necrosis factor alpha (TNF-α) in hippocampus were assessed by Western blot.

**Results:**

Surgery impaired learning and memory, and it increased expression of CXCL13 and CXCR5 in the hippocampus. CXCL13 knockdown partially reversed the effects of surgery on CXCR5 and cognitive dysfunction. CXCR5 knockout led to similar cognitive outcomes as CXCL13 knockdown, and it repressed surgery-induced activation of ERK and production of IL-1β and TNF-α in hippocampus. Recombinant CXCL13 induced cognitive deficits and increased the expression of phospho-ERK as well as IL-1β and TNF-α in hippocampus of wild-type mice, but not *CXCR5*^−/−^ mice. PD98059 partially blocked CXCL13-induced cognitive dysfunction as well as production of IL-1β and TNF-α.

**Conclusions:**

CXCL13-induced activation of CXCR5 may contribute to PNDs by triggering ERK-mediated production of pro-inflammatory cytokines in hippocampus.

## Background

Perioperative neurocognitive disorders (PNDs) refer to pre- and post-operative reduction in hippocampus-dependent cognitive functions involving disturbance of memory, consciousness and attention, as well as changes in personality [1, 2]. PNDs are a well-recognized clinical complication and patients of any age undergoing surgical procedure are susceptible. Among patients undergoing major noncardiac surgery, PNDs occur before discharge in 37% of patients aged 18–39 years, 30% of patients aged 40–59, and 41% of patients 60 years or older [3]. PNDs not only increase the burden on the healthcare system but also lead to postoperative long-term disability and even mortality. The use of volatile anesthetics during surgery has long been suspected of increasing risk of PNDs [4, 5]. As the number of surgeries continues to increase, PNDs are expected to become an epidemic [1]. Increasing evidence suggests that PNDs involve neuroinflammation in the hippocampus, at least in their early stages [2, 4]. Under certain conditions, secreted chemokines contribute to neuroinflammation in the central nervous system (CNS) [6–8]. Whether and how chemokines may contribute to PNDs is unclear, particularly in the hippocampus.

C-X-C motif chemokine (CXCL) 13 recruits B cells to the CNS in different neuroinflammatory diseases [6]. C-X-C motif chemokine 13 (CXCL13) helps mediate the inflammatory response to infection and injury, and it exerts local, concentration-dependent effects on hippocampal memory function [9]. Elevated levels of CXCL13 have been reported in the cerebrospinal fluid of patients with multiple sclerosis or neuromyelitis optica, and it has even been proposed as a biomarker for these neurodegenerative conditions [7]. The only known CXCL13 receptor is C-X-C motif chemokine receptor 5 (CXCR5), which is highly expressed on B lymphocytes and certain T cells in cerebrospinal fluid, and which helps guide lymphocyte tracking within secondary lymphoid tissues [10–13]. Our previous work showed that CXCR5 contributes to learning and memory impairment in an animal model of sepsis [14]. CXCR5 is expressed in neuronal precursor cells of the brain, which migrate across brain endothelial cells upon exposure to CXCL13 [11]. CXCL13/CXCR5 signaling in the spinal cord mediates neuroinflammation induced by neuropathic pain [13]. Expression of CXCL13 and CXCR5 is altered in intractable temporal lobe epilepsy patients and epileptic rats that show neurodegeneration and cognitive impairment [9]. These considerations led us to ask whether CXCL13 and CXCR5 are involved in PNDs.

We began to investigate this question by focusing on extracellular signal-regulated kinase (ERK). The CXCL13/CXCR5 axis acts via ERK to trigger production of pro-inflammatory cytokines induced by orofacial neuropathic pain [13], and CXCL13 appears to activate ERK by triggering its phosphorylation to p-ERK [15, 16]. ERK is highly expressed in the neurons of the developing and adult brain, functioning as an important mediator of synaptic plasticity and cell survival [17]. Changes in hippocampal levels of p-ERK have been linked to cognitive impairment in a mouse model of Alzheimer’s disease [18], and p-ERK appears to induce production of pro-inflammatory cytokines, such as tumor necrosis factor alpha (TNF-α) and interleukin-1 beta (IL-1β), in animal models of cognitive deficits [19].

The purpose of the present study was to examine whether CXCL13 may contribute to PNDs in an animal model, and more concretely whether it does so via ERK.

## Methods

### Animals

The animal protocol was approved by the Institutional Animal Ethics Committee of Nanjing Medical University. All experiments described here were conducted in strict accordance with institutional guidelines.

Male C57BL/6J wild-type (WT) mice (3 months old, 33–40 g) were obtained from the Experimental Animal Center of Nanjing First Hospital. *CXCR5*^*−*/*−*^ mice [B6.129S2 (Cg)-*CXCR5* ^tm1Lipp/J^, stock number 006659] bred on a B6 background were kindly provided by Professor Yongjing Gao (Pain Research Laboratory, Nantong University, China) [13]. WT and *CXCR5*^−/−^ mice were age-, gender-, and weight-matched. All mice were housed in a pathogen-free facility in the Experimental Animal Center at Nanjing First Hospital and maintained under a 12-h light-dark cycle with free access to food and water.

### Experimental design

**Fig. 1:**
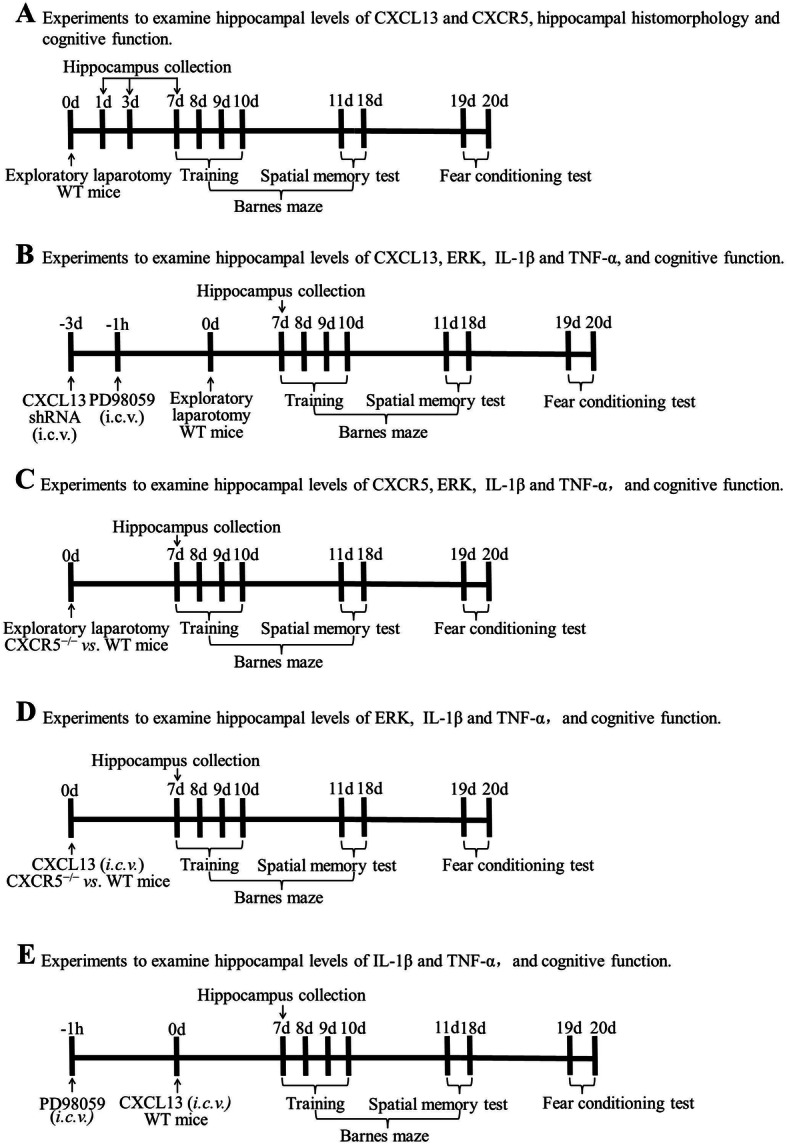
Schematic illustration of the experimental design. *CXCL13* C-X-C motif chemokine 13, *CXCR5* C-X-C motif chemokine receptor 5, *HE* hematoxylin-eosin staining, *i.c.v.* intracerebroventricular, *IL*-*1β* interleukin-1 beta, *TNF*-*α* tumor necrosis factor alpha, *WT* wide-type

The overall experimental design is summarized in Fig. 1. The mouse PND model was established using exploratory laparotomy. All pharmacological treatments were given via intracerebroventricular (i.c.v.) injection into the left ventricle. Several experimental procedures with different timelines were performed in order to comprehensively assess the roles of CXCL13, CXCR5, and ERK in PNDs. The following is a brief description of the experimental groups, and their manipulations are described in detail in subsequent subsections. (1) A cohort of WT mice was divided into two groups, one of which underwent laparotomy (*n* = 8) and the other of which did not (*n* = 8; termed controls). No additional pre-treatments were administered. (2) A cohort of WT mice was divided to randomly receive short hairpin RNA (shRNA) targeting CXCL13 or scrambled control shRNA at 3 days before surgery (*n* = 8). Additionally, ERK inhibitor PD98059 or vehicle was injected 1 h before surgery (*n* = 8). (3) A cohort of WT and *CXCR5*^−/−^ mice was randomly selected to undergo surgery without prior treatment (*n* = 8 of each strain). (4) A cohort of WT and *CXCR5*^−/−^ mice that did not undergo surgery was injected i.c.v. with recombinant murine CXCL13 (to mimic surgical insult) or with vehicle (*n* = 8). (5) A cohort of WT mice was injected with ERK inhibitor PD98059 at 1 h before treatment with recombinant CXCL13 (*n* = 8).

### Exploratory laparotomy model

Exploratory laparotomy was performed on mice in a sterile environment as described [20], with some modifications. To ensure a similar degree of stimulation, every mouse was explored using the same set of procedures. Briefly, mice were anesthetized by 1.5% isoflurane inhalation and kept under spontaneous respiration with an inspiration of 50% O_2_. Temperature was monitored rectally (Techman Software, Chengdu, China) and maintained at 37 ± 0.5 °C with the aid of a heating blanket. To mimic clinical exploratory laparotomy, a longitudinal midline incision was made in the abdomen from the xiphoid process to the superior margin of the pubic symphysis. A cotton tip wetted with 0.9% sterile saline (Baxter Healthcare, Shanghai, China) explored abdominal organs in the following order: liver, spleen, left and right kidneys, bladder and bowel. The incision site was infiltrated with bupivacaine (0.25%, 3 mg/kg) for postoperative analgesia. Then, the peritoneum and skin were sutured separately. The total duration of exploration and anesthesia lasted around 2 h. After animals recovered, they were returned to their cages with food and water ad libitum.

### Pharmacological treatments

All drugs were prepared under sterile conditions. The dose for i.c.v. injection was 5 μg for PD98059 (Sigma, St. Louis, MO, USA) [17] as well as recombinant murine CXCL13 (catalog no. 250-24, PeproTech, Rocky Hills, NJ, USA) [13]. The final DMSO concentration was 2.5%. Vehicle solution was 2.5% DMSO. Solutions were administered by i.c.v. injection. An shRNA targeting CXCL13 was expressed from the sequence 5′-TCG TGC CAA ATG GTT ACA A-3′ in a plasmid (GenePharma, Shanghai, China) [13], while negative control scrambled shRNA was expressed from the sequence 5′-TTC TCC GAA CGT GTC ACG T-3′. The shRNAs (5 μg) were dissolved in 5 μL RNase-free water, mixed with 5 μL transfection reagent (Entranster™-in vivo, Engreen Biosystem, Beijing, China), and injected i.c.v. at 3 days before exploratory laparotomy as described previously [14]. CXCL13 knockdown was verified using Western blot analysis.

### Intracerebroventricular injections

Mice were anesthetized with 1.5% isoflurane inhalation, and placed in a stereotaxic apparatus (Stoelting, Wood Dale, IL, USA). I.c.v. injection was performed using a 32-gauge microsyringe (Hamilton, Reno, NV, USA) over a period of 10 min, and the microsyringe was left in place for 5 min after completing the infusion. The stereotaxic coordinates were determined as 1.0 mm to the left from midline, 0.5 mm posterior to the bregma, and 2.5 mm below the dural surface [21]. The animals were taken to the individual cages after the *i.c.v.* injection.

### Barnes maze test

The Barnes maze trial was performed to assess the spatial learning and memory of mice as described [22], with modifications. Animals were placed in the middle of a round platform with 20 equal-sized peripheral holes spaced at regular intervals. One of the holes, called the “target hole,” was connected to a dark box. After the mouse was placed in the middle of the platform, aversive noise (85 dB) and bright light from a 200-W bulb were used to stimulate mice to seek and enter the target hole. Mice were trained in a spatial acquisition phase (3 min per trial, 2 trials each day at a 2-h interval for 4 days) beginning on day 7 after surgery. The spatial memory test was conducted 1 day after training ended (day 11 post-surgery) to measure short-term retention and 8 days after training (day 18 after surgery) to measure long-term retention. Mouse activity and the latency to find the target hole during each trial were recorded using the ANY-Maze video tracking system (SD Instruments, San Diego, CA, USA).

### Fear conditioning test

A fear conditioning test was conducted as described [23] on days 19 and 20 after surgery. All mice were placed in a chamber with a floor of stainless steel bars (LE111, Panlab, Barcelona, Spain) that had been wiped down with 70% ethanol. This chamber was designed to allow sound stimulation for the training of conditioned reflexes, as well as electrical stimulation on the feet using a constant-current generator. Mice were allowed to explore and habituate in the training environment for 3 min. Then they were stimulated with a monofrequency sound for 30 s (1 kHz, 80 dB), and during the last 2 s of this sound, a foot shock (1 mA) was delivered. After a 30-s pause, this pairing of sound and shock were delivered a second time. After 24 h, as a test of contextual memory, mice were allowed to enter the original chamber without any stimulation, and freezing behavior, defined as the absence of any movement other than that required for respiration for more than 3 s, was monitored using a SMART3.0 video tracking system (Panlab). Then, 2 h later, a cued fear conditioning test was conducted in which the animals were placed in the chamber and subjected to the monofrequency sound for 3 min without foot shock, and freezing behavior was monitored and recorded.

### Western blot

Hippocampus tissues were homogenized and centrifuged at 13,000 g at 4 °C for 10 min. The supernatant was separated on SDS-PAGE gels, and then transferred to a nitrocellulose membrane (Hybond-ECL, Amersham Biosciences, Little Chalfont, UK). Membranes were blocked, then incubated overnight at 4 °C with antibodies against CXCL13 (1:100; Novus Biologicals, Littleton, CO, USA) or CXCR5 (1:1000; Santa Cruz Biotechnology, Santa Cruz, CA, USA); or antibodies against ERK (1:500), p-ERK (1:500), IL-1β (1:1000), or TNF-α (1:500) (Cell Signaling, Beverly, MA, USA). Next, blots were incubated with secondary antibodies (Sigma-Aldrich) conjugated to horseradish peroxidase. Protein bands were detected using enhanced chemiluminescence (Amersham Biosciences) and quantitated using Quantity One software (Bio-Rad Laboratories, Hercules, CA, USA). Band intensities were normalized to that of β-actin.

### Reverse transcription-quantitative polymerase chain reaction

Total RNA was extracted from hippocampal tissue using Trizol (Invitrogen, Carlsbad, CA, USA). Total RNA (1 μg) was reverse-transcribed using a reverse transcription kit (no. DRR047A; Takara Bio, Otsu, Japan) according to the manufacturer’s protocol. Reverse transcription-quantitative polymerase chain reaction (RT-qPCR) was performed using SYBR Green I dye detection (Takara Bio) in a Real-Time Detection system (Bio-Rad Laboratories). The following primers were used [13]: CXCL13 forward, 5′-GGC CAC GGT ATT CTG GAA GC-3′; CXCL13 reverse, 5′-ACC GAC AAC AGT TGA AAT CAC TC-3′; CXCR5 forward, 5′-TGG CCT TCTA CAG TAA CAG CA-3′; CXCR5 reverse, 5′-GCA TGA ATA CCG CCT TAA AGG AC-3′; GAPDH forward, 5′-GCT TGA AGG TGT TGC CCT CAG-3′; and GAPDH reverse, 5′-AGA AGC CAG CGT TCA CCA GAC-3′. PCR conditions included an initial step at 95 °C for 3 min, followed by 30 cycles at 95 °C for 1 min, 56 °C for 40 s, and 72 °C for 1 min. Melting curves were generated to verify amplification specificity. Quantification was performed using the 2-^ΔΔCT^ method, and cycle threshold values were normalized to those of GAPDH.

### Hematoxylin and eosin staining

Mice were perfused with 20-mL normal saline, followed by 20-mL 4% formaldehyde. Brain tissues were harvested and post-fixed with 4% paraformaldehyde at room temperature overnight and then embedded in paraffin. A series of 5-μm-thick coronal sections were deparaffinized, rehydrated, and stained by hematoxylin-eosin (HE). The stained sections were observed under an optical microscope (Olympus BX53, Tokyo, Japan) in order to assess histopathology in the hippocampal region.

### Immunohistochemical staining

Brain tissue was immunostained as described [24]. It was isolated, fixed in 4% paraformaldehyde, placed into paraffin blocks, and cut coronally into 6-μm sections. Sections were deparaffinized, rehydrated, heated at 95 °C for 15 min for antigen retrieval, then incubated in 3% H_2_O_2_ for 25 min at room temperature to block endogenous peroxidases. After blocking by goat serum for 30 min, the sections were incubated overnight at 4 °C with primary antibodies against CXCL13 (1:100; ab272874, Abcam, Cambridge, UK) or CXCR5 (1:100; ab133706, Abcam) or p-ERK (1:250; 4695, CTS, Danvers, MA, USA). Sections were then incubated with secondary antibodies for 60 min at room temperature. A 3,3′-diaminobenzidine kit (Bioss, Beijing, China) was used to visualize antibody binding, and the sections were counterstained with hematoxylin. Photomicrographs were captured by a digital camera connected to a microscope (3D HISTECH, Budapest, Hungary). The immunoreactive cells in CA1, CA3, and dentate gyrus of hippocampus were counted using Image Pro-Plus 6.0 (Media Cybernetics, Bethesda, MD, USA) in three randomly selected fields on each section.

### Statistical analysis

Statistical analysis was carried out using GraphPad Prism Version 5.01 (Graph Pad Software, San Diego, CA, USA). Continuous variables were assessed using Shapiro-Wilk test for distribution. The analysis showed normal distribution for all variables. Thus, all continuous variables are shown as mean ± standard deviation (SD). Inter-group differences in Barnes maze training results were assessed for significance using two-way repeated-measures ANOVA, followed by the Bonferroni post hoc multiple comparison test. Inter-group differences on other variables were assessed using Student’s *t* test (for comparisons between two groups) or one-way ANOVA followed by Tukey’s multiple test (for comparisons among at least three groups). *P* < 0.05 was considered statistically significant.

## Results

### Surgery in mice impairs cognitive function and hippocampal morphology as a model of PNDs

**Fig. 2:**
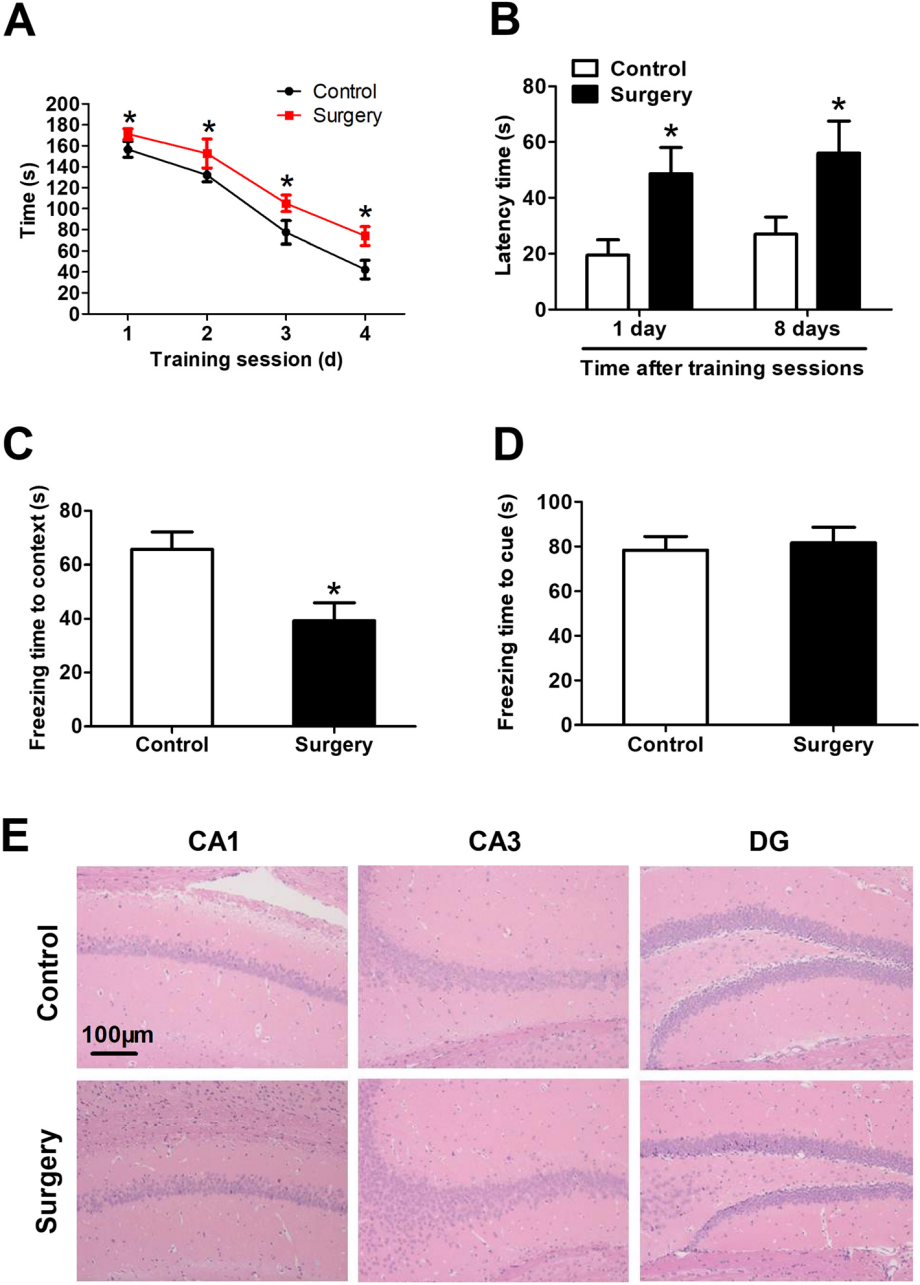
Surgery in mice impairs cognitive function and hippocampal morphology as a model of PNDs. Adult male C57BL/6J mice were subjected to exploratory laparotomy (termed “Surgery”). Control mice did not undergo surgery. All mice were subjected to 4 days of training in the Barnes maze beginning 7 days after the surgery; memory phase testing was performed on days 11 and 18 post-surgery. **a** Time to find the target hole during the training sessions and **b** latency in identifying the target hole after training were measured. Fear conditioning tests were performed on days 19 and 20 after surgery. Freezing times were quantified **c** in context and **d** in response to a cue (*n* = 8 per group). **e** Micrographs of hippocampal sections stained with hematoxylin and eosin (*n* = 4 per group). Magnification, × 20 for regions CA1, CA3, and DG in hippocampus. *P < 0.05 vs. control

Times in the Barnes maze decreased over the four training sessions in both control and surgery groups, and the mice that underwent surgery took longer to identify the target hole than controls (Fig. 2a). Surgery significantly prolonged latency time to identify the target hole at 1 or 8 days after training (Fig. 2b). Surgery shortened freezing times in the contextual fear conditioning test (Fig. 2c) without affecting freezing times in the cued fear conditioning test (Fig. 2d). HE staining also showed that surgery resulted in irregular neuronal arrangement, loss of morphology, and increases in neuronal swelling, nuclear pyknosis, and cellular vacuolization in the hippocampus (Fig. 2e).

### Surgery upregulates CXCL13 in mouse hippocampus

**Fig. 3:**
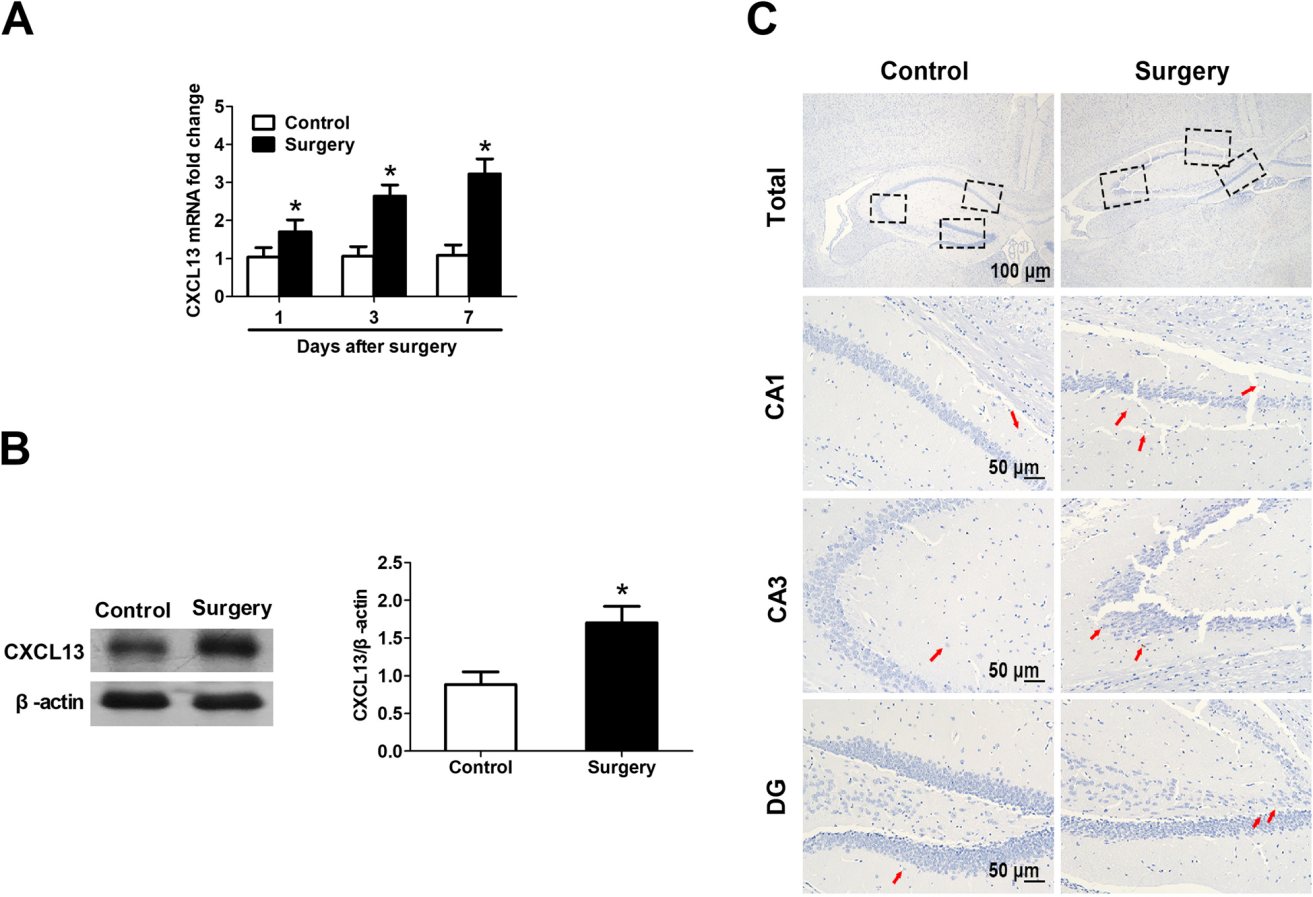
Surgery up-regulates CXCL13 in mouse hippocampus. **a** Relative level of CXCL13 mRNA was measured by RT-qPCR in wild type mice at 1, 3, and 7 days after surgery. **b** Western blotting analysis of CXCL13 at 7 days after surgery. β-actin was used as an internal control (*n* = 8 per group). **c** Representative images of CXCL13 immunohistochemistry in the hippocampal CA1, CA3, and dentate gyrus regions. Positively stained cells are brown (red arrow). Magnification, × 5 for total hippocampus; × 20 for regions CA1, CA3, and DG in hippocampus. All experiments were performed with *n* = 4 per group. *P < 0.05 vs. control. *CXCL13* C-X-C motif chemokine 13, *DG* dentate gyrus, *RT*-*qPCR* reverse transcription-quantitative polymerase chain reaction

We checked CXCL13 mRNA levels in the hippocampus on days 1, , and 7 after surgery. Tissue from surgically treated mice had higher CXCL13 mRNA levels than control tissue at all timepoints (Fig. 3a). CXCL13 protein expression was higher in surgically treated animals than in controls on day 7 after surgery (Fig. 3b). Immunohistochemical staining of hippocampus showed more CXCL13-expressing neurons in the CA1, CA3, and dentate gyrus at 7 days after surgery than in controls (Fig. 3c).

### CXCL13 knockdown alleviates PND-like cognitive deficits in mice

**Fig. 4:**
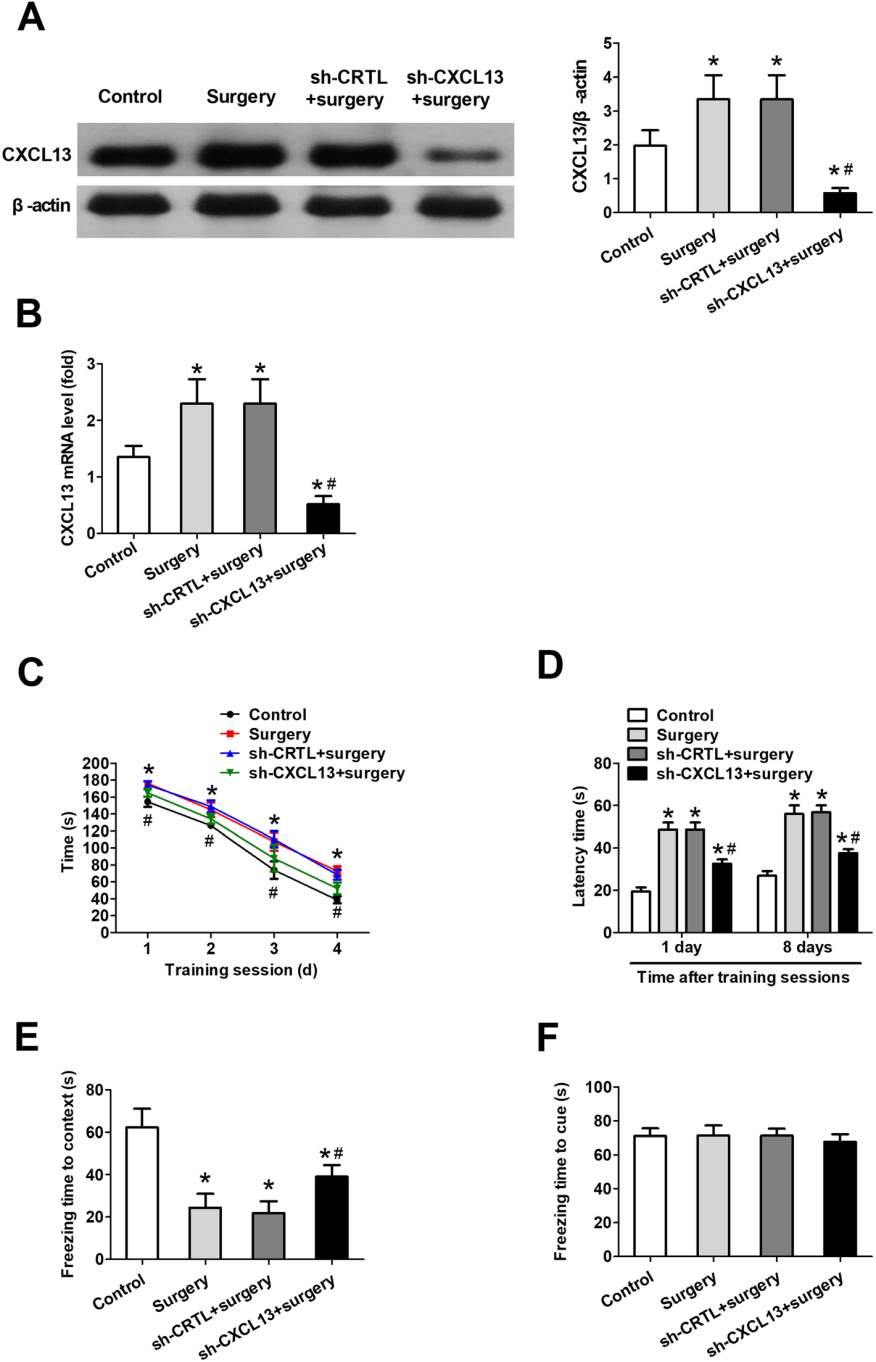
CXCL13 knockdown alleviates PND-like cognitive deficits in mice. Mice were pretreated with CXCL13 shRNA or scrambled shRNA, subjected to surgery and then behavioral tests were performed 7 days after surgery. **a** Western blotting and densitometry of hippocampal CXCL13, as well as **b** RT-qPCR analysis of the corresponding mRNA. **c** Time to identify the target hole in training sessions and **d** latency time during memory phases were quantified in the Barnes maze. In the fear conditioning tests, freezing times were quantified **e** in context and **f** in response to a cue. All quantitative results (mean ± SD) shown were obtained with *n* = 8 animals per group. *P < 0.05 vs. control; ^#^*P* < 0.05 vs. surgery. *CXCL13* C-X-C motif chemokine 13, *RT*-*qPCR* reverse transcription-quantitative polymerase chain reaction, *shRNA* short hairpin RNA, *sh*-*CRTL* scrambled shRNA, *sh*-*CXCL13* CXCL13 shRNA

Mice that underwent surgery alone showed higher CXCL13 expression than controls (Fig. 4 a, b). To examine the role of CXCL13 in the development and maintenance of cognitive dysfunction, we injected CXCL13 shRNA into the lateral ventricle 3 days before surgery. CXCL13 knockdown significantly reduced CXCL13 expression in surgically treated mice, while scrambled RNA had no effect on CXCL13 expression (Fig. 4 a, b). Time to identify the target hole in the Barnes maze trial progressively decreased over the 4 days of training sessions in all groups (Fig. 4c). All surgically treated groups took significantly longer to find the target hole than the control group during training and testing, regardless of CXCL13 levels (Fig. 4 c, d ). However, mice transfected with CXCL13 shRNA prior to surgery identified the target hole faster than animals treated with surgery alone or with surgery and scrambled shRNA.

All mice that underwent surgery exhibited shorter freezing times than controls in the contextual fear conditioning test (Fig. 4e), and CXCL13 knockdown partially reversed this surgery-induced impairment. In contrast, the groups did not differ significantly in freezing times in the hippocampal-independent cued fear conditioning test (Fig. 4f).

### Surgery upregulates CXCR5 in mouse hippocampus

**Fig. 5:**
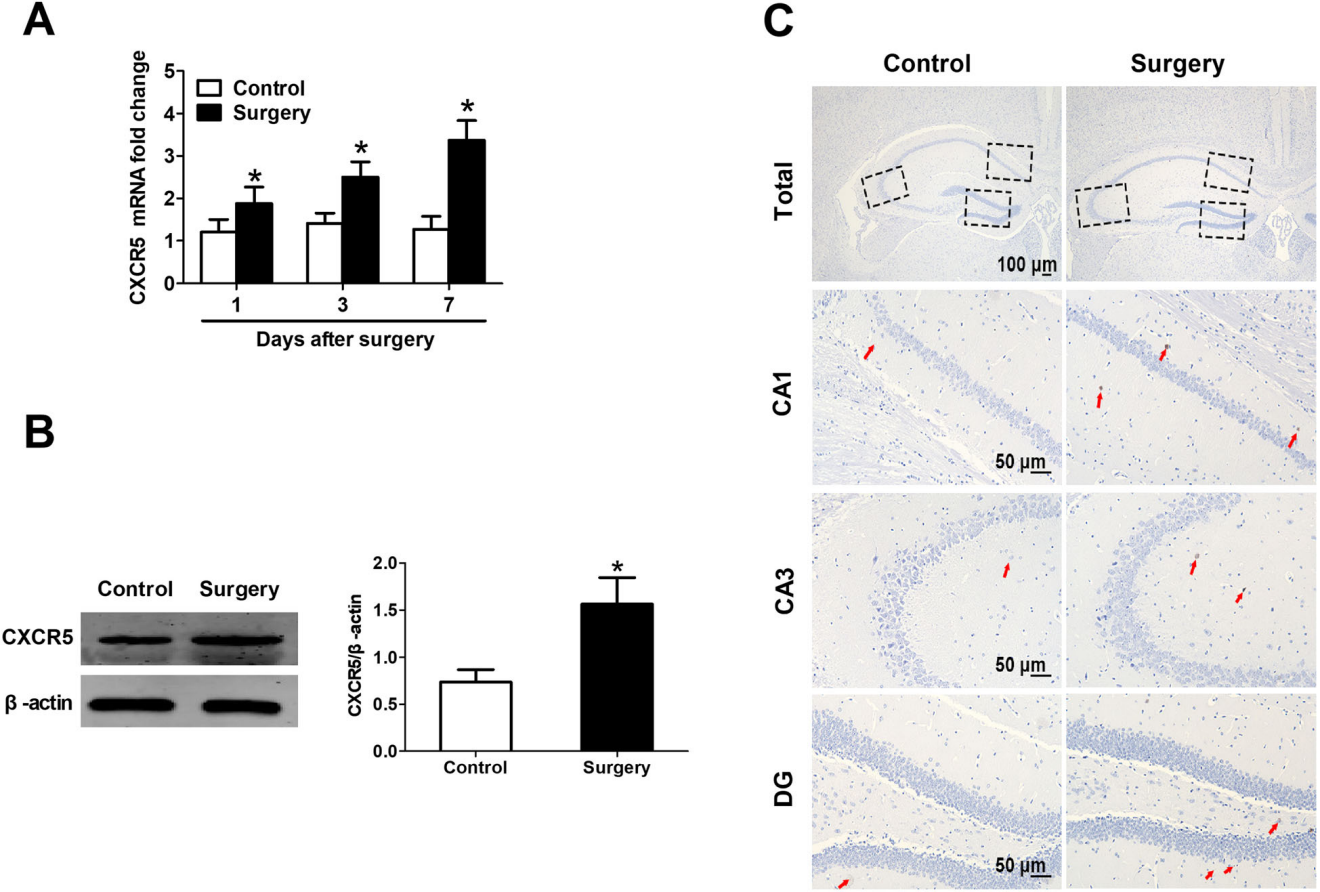
Surgery up-regulates CXCR5 in mouse hippocampus. **a** Relative level of CXCR5 mRNA was measured by RT-qPCR on days 1, 3, and 7 after surgery. **b** Western blotting analysis and densitometry of CXCR5 protein levels. β-actin was used as an internal control (*n* = 8 per group). **c** Representative images of CXCR5 immunohistochemistry in the hippocampal CA1, CA3, and dentate gyrus. Positively stained cells are brown (red arrow). Magnification, × 5 for total hippocampus; × 20 for hippocampal CA1, CA3, and DG (*n* = 4 per group). *P < 0.05 vs. control. *CXCR5* C-X-C motif chemokine receptor 5, *DG* dentate gyrus, *RT*-*qPCR* reverse transcription-quantitative polymerase chain reaction

CXCR5 mRNA levels were higher in hippocampal tissue at 1, 3, and 7 days after surgery (Fig. 5a). Similarly, the level of corresponding protein was higher in surgically treated mice than controls at 7 days after surgery, as shown by Western blot and immunohistochemistry (Fig. 5 b, c ).

### Deletion of CXCR5 alleviates PND-like cognitive deficits in mice

**Fig. 6:**
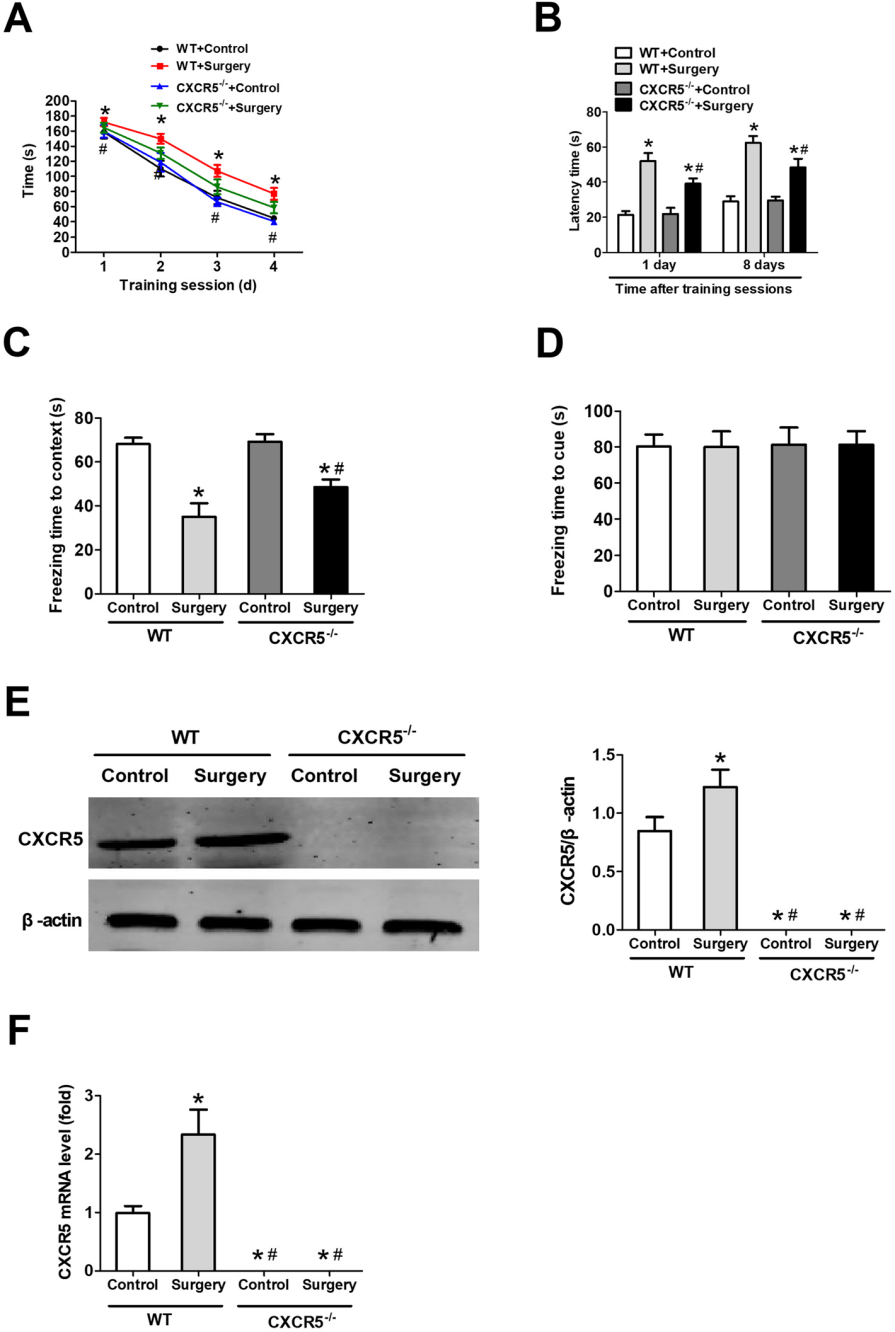
Deletion of *CXCR5* alleviates PND-like cognitive deficits in mice. WT or mice lacking the *CXCR5* gene (*CXCR5*^−/−^) were subjected to surgery (denoted as “surgery”). Mice that did not undergo surgery were denoted “control.” All mice were tested in the Barnes maze and fear conditioning tests at 7 days after surgery. **a** Time to identify the target hole during training sessions and **b** latency time during memory phases of the Barnes maze. In the fear conditioning test, freezing times were quantified **c** in context and **d** in response to a cue. **e** Western blot and densitometry of hippocampal CXCR5, as well as **f** RT-qPCR analysis of the corresponding mRNA. All quantitative results (mean ± SD) shown were obtained with *n* = 8 animals per group. *P < 0.05 vs. WT + control; ^#^*P* < 0.05 vs. WT + surgery. *CXCR5* C-X-C motif chemokine receptor 5, *RT*-*qPCR* reverse transcription-quantitative polymerase chain reaction, *WT* wild-type

While all groups became increasingly faster with each training session in the Barnes maze trial, surgery impacted time more than CXCR5 levels (Fig. 6a). Surgically treated *CXCR5*^*−/−*^ mice identified the target hole faster than surgically-treated WT mice (Fig. 6b).

Although all mice that underwent surgery exhibited shorter freezing times than control mice in the contextual fear conditioning test, no differences were seen among the groups during the cued fear conditioning test (Fig. 6 c, d ). Surgically treated WT mice had higher CXCR5 mRNA and protein expression than control WT mice (Fig. 6 e, f ).

### CXCR5 deficiency inhibits surgery-induced p-ERK activation and release of inflammatory cytokines in mouse hippocampus

**Fig. 7:**
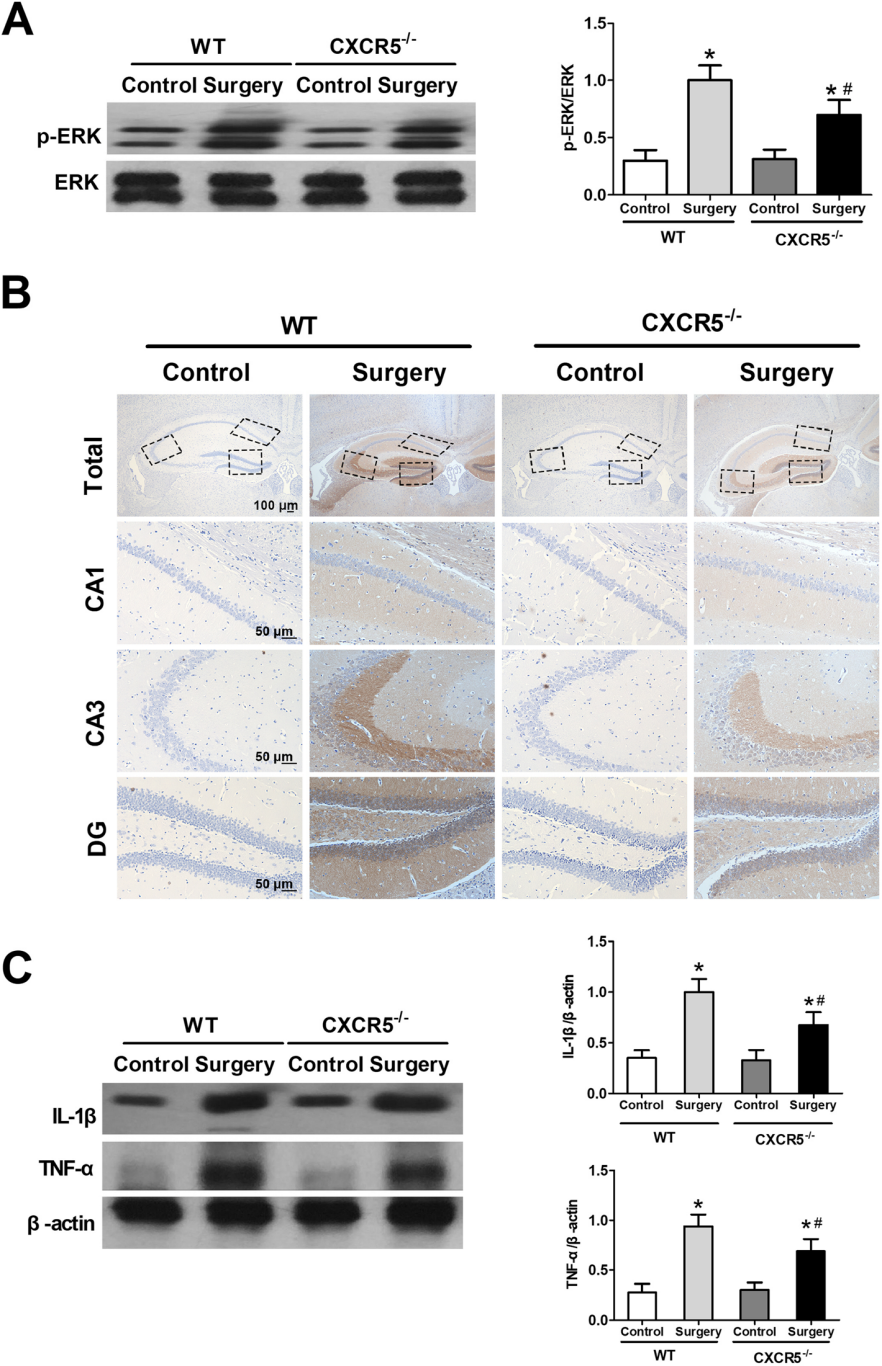
CXCR5 deficiency inhibits surgery-induced p-ERK activation and release of inflammatory cytokines in mouse hippocampus. **a** Western blotting analysis of total ERK and p-ERK (*n* = 8 per group). **b** Representative images of p-ERK immunohistochemistry in the hippocampus. Positive cells are stained brown. Magnification, × 5 for total hippocampus; × 20 for hippocampal CA1, CA3, and DG (*n* = 4 per group). **c** Western blot and densitometry of IL-1β and TNF-α in the hippocampus. β-actin was used as an internal control (*n* = 8 per group). *P < 0.05 vs. control; ^#^*P* < 0.05 vs. surgery. *CXCR5* C-X-C motif chemokine receptor 5, *DG* dentate gyrus, *WT* wild-type

WT and *CXCR5*^−/−^ mice with surgery had significantly higher p-ERK expression in the hippocampus on day 7 after surgery than counterparts that did not undergo surgery (Fig. 7a). However, total ERK levels were unaffected. CXCR5 knockout reduced p-ERK levels in surgically treated mice but not control mice. Moreover, surgery induced an increase in the number of p-ERK-containing neurons in the hippocampus of WT mice on day 7, and less so in *CXCR5*^−/−^ mice (Fig. 7b). There was no difference in the number of p-ERK-containing neurons in control animals of either genotype.

TNF-α and IL-1β are important pro-inflammatory cytokines in regulating cognitive dysfunction in the CNS [25]. We checked TNF-α and IL-1β expression in the hippocampus on day 7 after surgery. WT mice with surgery had higher TNF-α and IL-1β expression than controls (Fig. 7c). Surgically treated *CXCR5*^−/−^ mice had lower TNF-α and IL-1β expression than surgically treated WT mice.

### ERK-dependent production of pro-inflammatory cytokines in hippocampus mediates PND-like cognitive deficits in mice

**Fig. 8:**
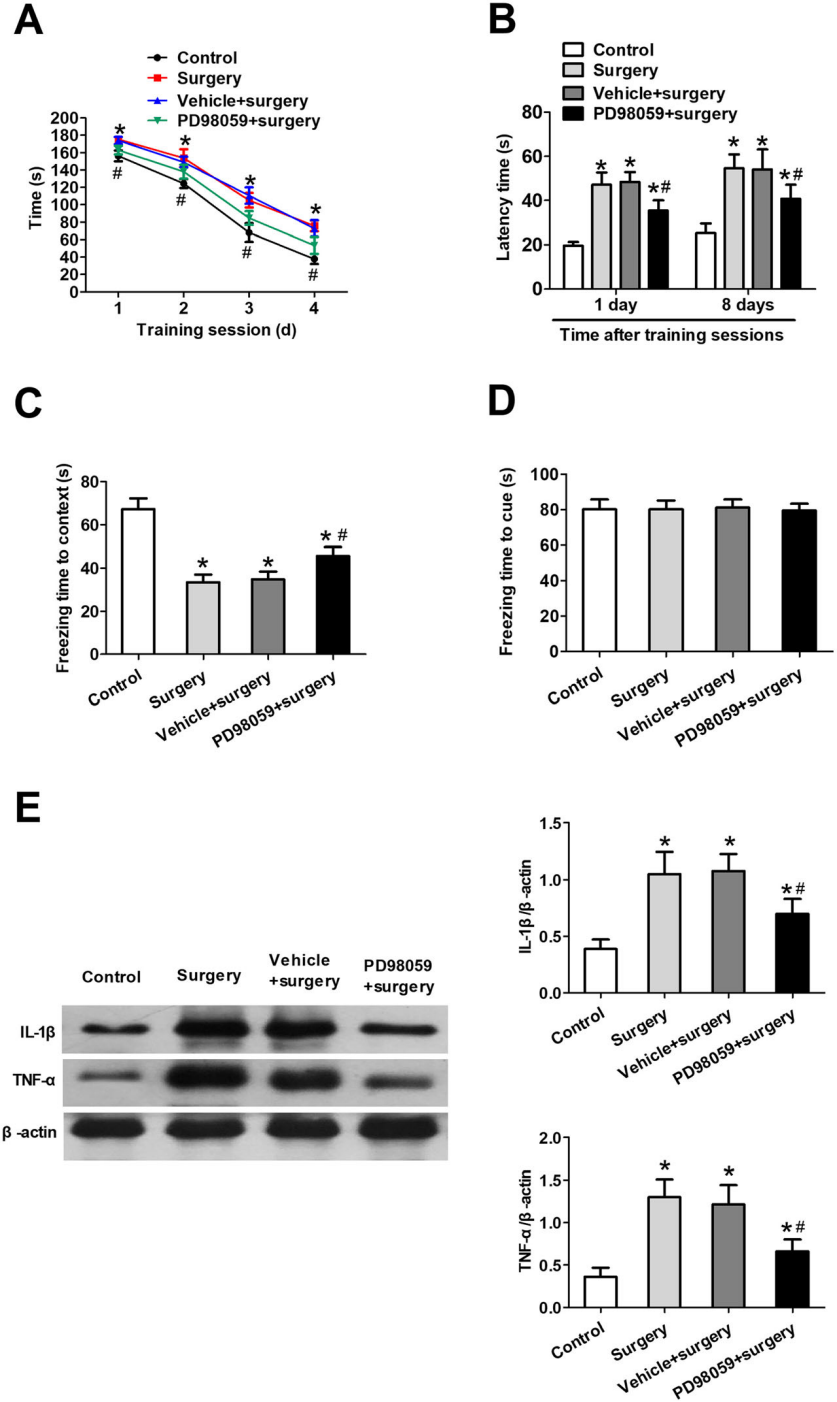
ERK-dependent production of pro-inflammatory cytokines in hippocampus mediates PND-like cognitive deficits in mice. Mice subjected to surgery were pretreated with vehicle or PD98059, and behavioral tests were conducted 7 days after the surgery. **a** Time to find the target hole during training sessions and **b** latency time during memory phases were assessed in the Barnes maze. In the fear conditioning test, freezing times were quantified **c** in context and **d** in response to a cue. **e** Western blot and densitometry of hippocampal IL-1β and TNF-α protein levels. β-actin was used as an internal control. All quantitative results (mean ± SD) shown were obtained with *n* = 8 animals per group. *P < 0.05 vs. control; ^#^*P* < 0.05 vs. surgery. *TNF*-*α* tumor necrosis factor alpha, *IL*-*1β* interleukin-1 beta

Mice pretreated with PD98059 displayed shorter time to the target hole on all training days (Fig. 8a), and also had shorter latency than surgically-treated WT mice (Fig. 8b). PD98059 pretreatment also rescued PND-like effects in the contextual fear conditioning test (Fig. 8c), but made no difference in cued fear freezing (Fig. 8d). PD98059 pretreatment downregulated TNF-α and IL-1β expression in surgically treated mice (Fig. 8e).

### CXCL13 induces CXCR5/ERK-dependent release of pro-inflammatory cytokines in mouse hippocampus

**Fig. 9:**
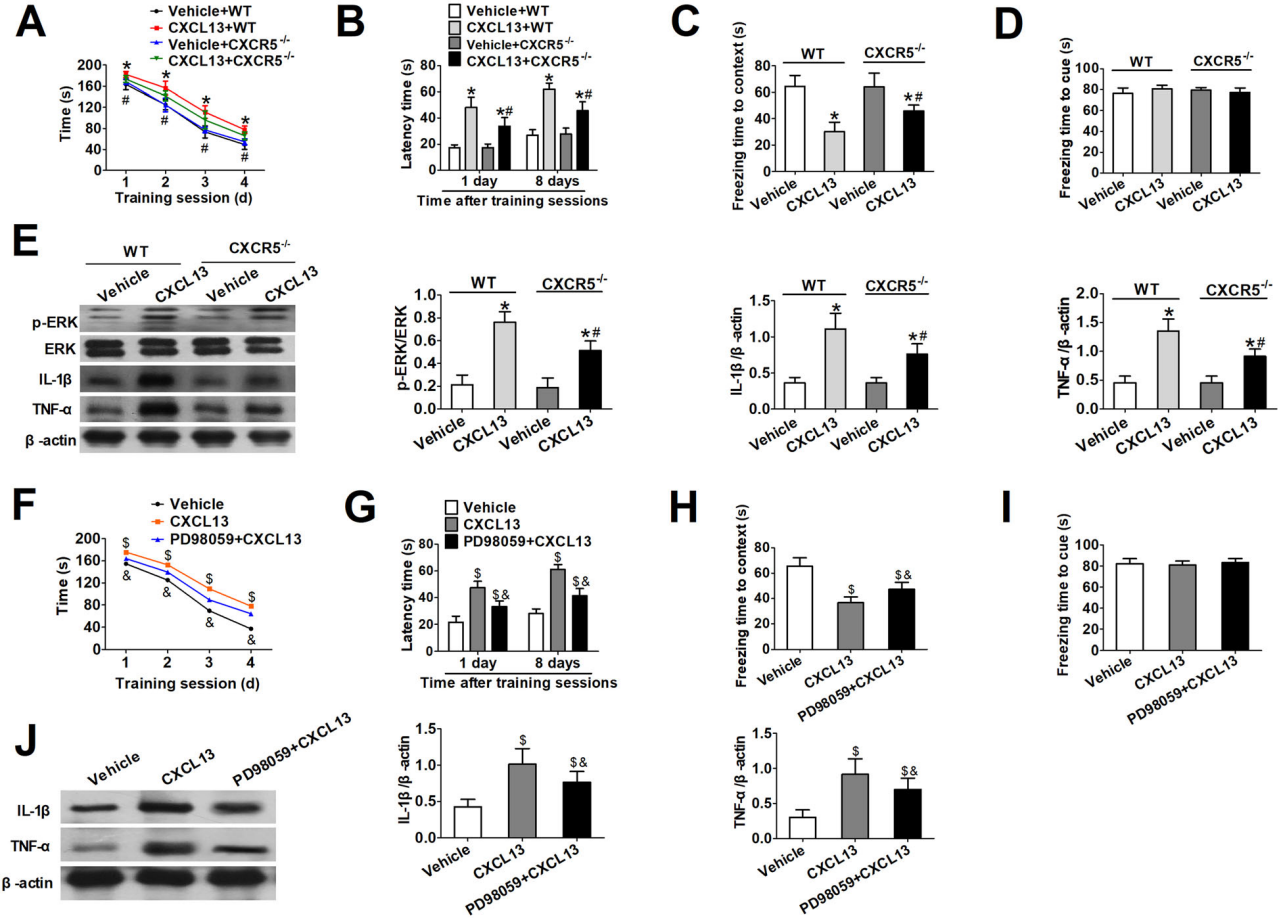
CXCL13 induces CXCR5/ERK-dependent release of pro-inflammatory cytokines in mouse hippocampus. WT or *CXCR5*^−/−^ mice received CXCL13 via intracerebroventricular injection. Seven days later, Barnes maze and fear conditioning tests were conducted. **a** Time to identify the target hole during training sessions and **b** latency time during memory phase were measured in the Barnes maze. Freezing times were quantified **c** in context and **d** in response to a cue. **e** Western blotting and densitometry of hippocampal p-ERK, total ERK, IL-1β, and TNF-α. Mice were injected with CXCL13 with or without PD98059 pretreatment, and behavioral tests were performed 7 days later. **f** Time to identify the target hole in training sessions and **h** latency time in memory phases of the Barnes maze. Freezing times were quantified **i** in context and **j** in response to a cue in fear conditioning tests. **k** Western blot and densitometry of IL-1β and TNF-α. All quantitative results (mean ± SD) shown were obtained with *n* = 8 animals per group. *P < 0.05 vs. vehicle + WT; ^#^*P* < 0.05 vs. CXCL13 + WT; ^$^*P* < 0.05 vs. vehicle; ^&^*P* < 0.05 vs. CXCL13. *CXCL13* C-X-C motif chemokine 13, *CXCR5* C-X-C motif chemokine receptor 5, *WT* wild-type, *TNF*-*α* tumor necrosis factor alpha, *IL*-*1β* interleukin-1 beta

Compared with control mice, all CXCL13 treatment groups took more time to identify the target hole across all days (Fig. 9 a, b ), and these impairments were partially reversed by deleting *CXCR5*. Additionally, CXCL13 treatment shortened freezing time in contextual fear conditioning, and it did so to a smaller extent in *CXCR5*^−/−^ mice (Fig. 9c). No differences were found in the cued fear conditioning test (Fig. 9d). CXCL13 increased the levels of p-ERK, IL-1β, and TNF-α, and the effect was mitigated by CXCR5 deficiency (Fig. 9e).

Pretreatment PD98059 partially attenuated CXCL13-induced cognitive dysfunction (Fig. 9 f–i ). Additionally, CXCL13 mice pretreated with PD98059 had lower expression of TNF-α and IL-1β than mice treated with CXCL13 alone (Fig. 9j).

## Discussion

The present study explored whether chemokine CXCL13 and its receptor CXCR5 are involved in surgery-induced cognitive dysfunction in an animal model of PND. Our data clearly demonstrate that surgical intervention induced CXCL13 release and an increase in CXCR5 expression followed by cognitive impairment as observed in behavioral tests, and that inhibition of CXCL13/CXCR5 signaling attenuated surgery-induced cognitive impairment. We also investigated the role of ERK in surgery-induced neuroinflammation. Surgery induced ERK activation and ERK-dependent production of the inflammatory factors IL-1β and TNF-α in the hippocampus of mice, but this effect was repressed by *CXCR5* knockout. Finally, i.c.v. injection of recombinant CXCL13 induced CXCR5/ERK-dependent upregulation of TNF-α and IL-1β as well as cognitive dysfunction. Altogether, our results suggest that CXCL13 induces PND, at least partially, by triggering hippocampal CXCR5-ERK signaling.

CXCL13, a member of the chemokine family, is the major determinant for B cell recruitment to the intrathecal compartment during a range of human neurological disorders [6, 26]. Studies in animals and humans suggest that CXCL13 contributes to certain CNS disorders, and that blockade of this ligand might have therapeutic effects [25]. CXCL13 has been proposed as a biomarker of neuroinflammatory disease due to its high level in the CNS under a variety of conditions, including multiple sclerosis and neuromyelitis optica [7] as well as CNS lymphoma [27, 28]. Whether CXCL13 is involved in the pathogenesis of PNDs remains unclear. In the present study, CXCL13 mRNA in the hippocampus was upregulated 1 day after surgery and remained elevated for more than 7 days in surgically treated mice with cognitive impairment; Western blotting and immunostaining further showed upregulated CXCL13 protein in the hippocampus. In agreement with our results, several reports showed that CXCL13 was increased in dorsal root ganglion in a neuropathic pain model and hippocampus in CNS infection [29–32], and its high expression was detected mainly in microglia, macrophages, and endothelial cells [13, 33]. To examine the role of CXCL13 in the development and maintenance of cognitive dysfunction, we knocked it down specifically using shRNA. Knockdown effectively attenuated surgery-induced cognitive dysfunction. These data suggest the involvement of hippocampal CXCL13 in surgery-induced PNDs.

CXCR5 is responsible for the organization of B cell follicles and for directing T-helper cells to the lymphoid follicles [34–37]. In the CNS, CXCR5 is expressed in neuronal precursor cells, which migrate across brain endothelial cells upon exposure to CXCL13 [11]. CXCL13 and CXCR5 are involved in neurodegeneration and cognitive decline [9], which are related to a deficit in hippocampal neurogenesis [38, 39]. In previous work, we showed that CXCR5 may inhibit proliferation, differentiation, and survival of hippocampal neuronal stem cells in an animal model of sepsis, leading to learning and memory impairments [14]. Hippocampal CXCR5 upregulation causes sevoflurane anesthesia-induced cognitive dysfunction via PI3K/Akt signaling [40]. In the present study, we found that surgery elevated hippocampal CXCR5 mRNA and protein levels for more than 7 days in mice. CXCR5 deficiency attenuated surgery-induced cognitive dysfunction. Additionally, i.c.v. injection of recombinant CXCL13 triggered learning and memory deficits in surgically treated animals, which were attenuated in *CXCR5*^−/−^ animals. These data suggest that CXCL13/CXCR5 signaling is necessary for the development and maintenance of PNDs.

CXCL13/CXCR5 signaling contributes to neurodegeneration in individuals with cognitive deficit disease [9], as well as in animal models with neuropathic pain [41]. In fact, animal studies have shown that CXCL13 and CXCR5 contribute to neuropathic pain via ERK signaling [13]. ERK is also activated in the hippocampus of animals anesthetized with sevoflurane or propofol, and these animals later show impaired learning and memory [42, 43]. Intra-prefrontal cortex administration of ERK inhibitor PD98059 in 6-month-old 3xTg mice reversed memory impairment, suggesting that ERK pathway alterations might at least partially explain memory deficits observed in an Alzheimer’s disease model [44]. Here, we found that surgery increased p-ERK levels in the hippocampus, and this upregulation was partially reversed by deleting *CXCR5*. Moreover, i.c.v. injection of CXCL13 induced CXCR5-dependent p-ERK upregulation in the hippocampus. Similarly, a recent study showed that CXCL13 activates ERK in the spinal cord through CXCR5 in a mouse model of diabetes-induced tactile allodynia [41]. These data suggest the important role of ERK in mediating CXCL13/CXCR5 signaling in the hippocampus.

Surgical trauma can cause neuroinflammation involving synaptic deficits, neuronal dysfunction and death, and impaired neurogenesis [45, 46]. ERK is known to mediate the expression of inflammatory mediators, including growth factors and pro-inflammatory cytokines (e.g., IL-1β) in an animal model of cognitive deficit [47]. IL-1β and TNF-α are important pro-inflammatory cytokines that mediate surgery-induced cognitive dysfunction [48]. We also found that surgery upregulated the pro-inflammatory cytokines IL-1β and TNF-α in the hippocampus, and this was partially reversed by deleting CXCR5 or knocking down ERK. Pretreatment with CXCL13 induced CXCR5/ERK-dependent release of pro-inflammatory cytokines in the hippocampus. Both CXCL13 and CXCR5 were upregulated in the brain in both patients with intractable epilepsy and a rat model of epilepsy with neuroinflammation [9]. Increased release of proinflammatory cytokines, including IL-1β and TNF-α, has been reported in the hippocampus of rodents after prolonged seizures [49], and contributes to cognitive impairment [50]. We did not observe spontaneous seizure in our experiments. Such a discrepancy warrants further investigation. Nevertheless, our findings data suggest that ERK-mediated release of pro-inflammatory cytokines in the hippocampus contributes to PNDs.

Previous studies showed that CXCR5^−/−^ mice have more immature neurons in the dentate gyrus, an increase in baseline locomotor activity, and decreased anxiety-like behavior [39]. CXCR5^−/−^ mice may also develop retinal degeneration with pathophysiological changes, such as activation of microglia and accumulation of inflammatory cells, loss of ZO-1 indicative of impaired blood-retinal barrier function after ischemia-reperfusion injury [51]. These changes could conceivably produce major impact on learning and memory, and thus bias the results in the current study.

## Conclusions

These results from the current study indicated that CXCL13 and CXCR5 contribute to PNDs, possibly by activating ERK to trigger release of pro-inflammatory cytokines in the hippocampus. Thus, targeting the CXCL13/CXCR5/ERK pathway in the hippocampus may provide a novel therapeutic approach to treat these disorders.

## Data Availability

All data generated or analyzed during this study are available from the corresponding author upon reasonable request.

## Abbreviations

CNS: Central nervous system
CXCL13: C-X-C motif chemokine 13
CXCR5: C-X-C motif chemokine receptor 5
ERK: Extracellular signal-regulated kinase
i.c.v.: Intracerebroventricular
IL-1β: Interleukin-1 beta
PND: Perioperative neurocognitive disorder
RT-qPCR: Reverse transcription-quantitative polymerase chain reaction
shRNA: Short hairpin RNA
TNF-α: Tumor necrosis factor alpha
WT: Wild-type
